# Higher Frequency Network Activity Flow Predicts Lower Frequency Node Activity in Intrinsic Low-Frequency BOLD Fluctuations

**DOI:** 10.1371/journal.pone.0064466

**Published:** 2013-05-15

**Authors:** Sahil Bajaj, Bhim Mani Adhikari, Mukesh Dhamala

**Affiliations:** 1 Department of Physics and Astronomy, Georgia State University, Atlanta, Georgia, United States of America; 2 Neuroscience Institute, Joint Center for Advanced Brain Imaging, Center for Behavioral Neuroscience, Georgia State University, Atlanta, Georgia, United States of America; University Of Cambridge, United Kingdom

## Abstract

The brain remains electrically and metabolically active during resting conditions. The low-frequency oscillations (LFO) of the blood oxygen level-dependent (BOLD) signal of functional magnetic resonance imaging (fMRI) coherent across distributed brain regions are known to exhibit features of this activity. However, these intrinsic oscillations may undergo dynamic changes in time scales of seconds to minutes during resting conditions. Here, using wavelet-transform based time-frequency analysis techniques, we investigated the dynamic nature of default-mode networks from intrinsic BOLD signals recorded from participants maintaining visual fixation during resting conditions. We focused on the default-mode network consisting of the posterior cingulate cortex (PCC), the medial prefrontal cortex (mPFC), left middle temporal cortex (LMTC) and left angular gyrus (LAG). The analysis of the spectral power and causal flow patterns revealed that the intrinsic LFO undergo significant dynamic changes over time. Dividing the frequency interval 0 to 0.25 Hz of LFO into four intervals slow-5 (0.01–0.027 Hz), slow-4 (0.027–0.073 Hz), slow-3 (0.073–0.198 Hz) and slow-2 (0.198–0.25 Hz), we further observed significant positive linear relationships of slow-4 in-out flow of network activity with slow-5 node activity, and slow-3 in-out flow of network activity with slow-4 node activity. The network activity associated with respiratory related frequency (slow-2) was found to have no relationship with the node activity in any of the frequency intervals. We found that the net causal flow towards a node in slow-3 band was correlated with the number of fibers, obtained from diffusion tensor imaging (DTI) data, from the other nodes connecting to that node. These findings imply that so-called resting state is not ‘entirely’ at rest, the higher frequency network activity flow can predict the lower frequency node activity, and the network activity flow can reflect underlying structural connectivity.

## Introduction

The brain consists of a collection of anatomically distinct and functionally relevant networks of brain regions [1], [2]. It is a self-organizing dynamical system [3] with ongoing neural oscillations coherent across distributed brain regions during resting state- under no explicit tasks or no external sensory stimulation [4], [5], [6]. The brain’s underlying structural connectivity determines the coherent neural activity. Recent neuroimaging studies provided evidence for the relationship between the underlying brain structural network (structure) and coherent oscillations (function) during resting conditions [7], [8], [9], [10], [11], [12]. However, the details of the relationship between the brain function and structure are still being revealed. Here, in this study, we evaluated how the frequency band-specific net information flow from a brain node correlates with the net anatomical connections to (or from) the node from BOLD fMRI and diffusion tensor imaging (DTI) data.

Even during resting conditions, networks of brain regions can be spontaneously active [13], [14], [15], [16], [17], [18]. These networks exhibit low-frequency (<0.1 Hz) oscillations or fluctuations (LFO) in functional magnetic resonance imaging (fMRI) blood-oxygen level dependent (BOLD) signals. These intrinsic BOLD oscillations are related with slow neuronal oscillations [19] possibly reflecting modulation of cortical excitability and long distance synchronization [20], [21], [22]. The level of these oscillations and the co-variation among the network nodes are temporally dynamic dependent on levels of awareness and arousal during resting conditions. A previous study by Chang and colleagues [23] suggested the dynamic nature of resting state functional connectivity. Despite tremendous progress in studying BOLD LFO across various brain regions, the dynamic nature of these fluctuations and the relationship with the underlying structural connectivity remains to be understood well. In the current study, without relying on the common temporal stationarity assumptions about BOLD LFO, we investigated the temporal dynamics of several frequency bands of BOLD LFO in the default-mode network (also known as task-negative network) comprising of the posterior cingulate cortex (PCC), the medial prefrontal cortex (mPFC), left middle temporal cortex (LMTC) and left angular gyrus (LAG). We then evaluated the structure-function relationship from DTI fiber tracts and the BOLD LFO in the network.

FMRI BOLD fluctuations recorded in eyes-open or eyes-closed resting conditions have provided opportunities to study the patterns of node and network-level activities in the brain. Jiao and colleagues [24] reported a linear relationship between node activity and network activity during eyes-closed resting conditions. Chang and Glover [23] reported a time-varying feature of resting-state functional connectivity. Zuo and colleagues [6] reported differential spatial distribution of four narrowly defined slow frequency bands of BOLD LFO within the brain in eyes-closed conditions. Here we evaluated time-varying activity of the default brain nodes and network, and the relationship between node activity and network in-out flow (net causal flow into a node) over various frequency bands of BOLD LFO with recently added Granger causality in the standard wavelet tools [25], [26]. We used the seed-based correlation approach [13], [27], and extracted time series from the seed node PCC (the posterior cingulate cortex) and other significantly positively correlated regions. In a seed-based correlation approach, a certain brain region is first chosen as a seed, and the average time-series of the seed region is used to compute cross-correlation coefficients with all other voxel time-series in the brain, and highly correlated voxels to the seed region are identified using a threshold for correlation value [13], [28]. PCC is a commonly selected seed region [23], [29], [30]. The PCC-correlated network is often assumed to be associated with mind wandering or day-dreaming [31], [32], [33], [34]. In our analysis, the PCC-correlated network involved these four nodes: the posterior cingulate cortex (PCC) (–6, –52, 40), medial prefrontal cortex (mPFC) (0, 48, –4), left middle temporal cortex (LMTC) (–57, –19, –11) and left angular gyrus (LAG) (–46, –64, 25). These co-ordinates are in the Montreal Neurological Institute (MNI) coordinate system. For the univariate and multivariate spectral analyses of the extracted BOLD fMRI time series from these nodes, we subdivided the frequency range 0 – 0.25 Hz into the following four bands as in the work of Zuo and colleagues [6] (i) slow-5: 0.01–0.027 Hz, (ii) slow-4: 0.027–0.073 Hz, (iii) slow-3: 0.073–0.198 Hz, and (iv) slow-2: 0.198–0.25 Hz. This division is based on the experimentally observed categories of neural oscillations on a log-scale with frequency [6], [20], [35].

In this study, we thus planned for the detailed spectral analysis of the characteristics of fMRI BOLD signals and the time-varying nature of the brain network activity during rest.

We evaluated the dynamic nature of default-mode networks, detailed frequency contents of low-frequency BOLD network oscillations, and its relationship of net information flow with the underlying anatomical connectivity.

## Materials and Methods

### Participants

A total of 49 healthy adults (28 males, 21 females) aged between 18–36 years underwent scanning. All participants provided written, informed consent. Georgia State University Institutional Review Board, and the Joint Institutional Review Board of Georgia State University and Georgia Institute of Technology, Atlanta approved experimental protocols. During functional MRI runs, participants were instructed to keep their eyes open fixated at the central cross on a screen, relax and try not to fall asleep. None of the participants in fMRI runs were found to move significantly or have fallen asleep. We had resting-state fMRI data from 17 participants (mean age: 25.17±4.68 years, 12 males, 5 females) and DTI data from 32 participants (mean age  = 27.7±5.17 years, 16 males, 16 females). There were 6 common participants who had both fMRI and DTI data. All 49 participants' imaging data (fMRI, DTI, or both) were included in the final analysis.

### Imaging

Magnetic resonance imaging was performed at two locations: at CABI (Georgia State and Georgia Tech Center for Advanced Brain Imaging, Atlanta) and BITC (Georgia Tech and Emory University Biomedical Imaging Technology Center, Atlanta) using 3-Tesla Siemens whole-body MRI scanners. Functional imaging was 7 minute and 54 sec long, and included a T2*-weighted echo planner imaging (EPI) sequence (echo time (TE)  = 40 ms; repetition time (TR)  = 2000 ms; flip angle  = 90^ο^; field of view (FOV)  = 24 cm, matrix  = 64×64; number of slices  = 33 and slice thickness  = 5 mm). High-resolution anatomical T1-weighted images were acquired for anatomical references using an MPRAGE sequence with an isotropic voxel size of 2 mm. Diffusion-weighted images (DTI) were acquired with 30 diffusion-encoding directions with an isotropic voxel size of 2 mm (b value  = 1000 s/mm^2^; 60 slices; TR  = 7700 ms; TE  = 90 ms; FOV read  = 204 mm, slice thickness  = 2 mm) plus one reference volume without diffusion weighting (b-value  = 0 s/mm^2^). DTI acquisition took approximately 10 minutes.

### FMRI Preprocessing

FMRI data were preprocessed by using SPM8 (Wellcome Trust Centre for Neuroimaging, London; http://www.fil.ion.ucl.ac.uk/spm/software/spm8/). The preprocessing steps involved slice time correction, realignment, normalization and smoothing. Motion correction to the first functional scan was performed within participant using a six-parameter rigid-body transformation. All participants included in this analysis had less than 2 mm of translation in all directions and less than 1.5° of rotation about the three axes. The mean of the motion-corrected images was then coregistered to the individual structural image using a 12-parameter affine transformation. The images were then spatially normalized to the Montreal Neurological Institute (MNI) template [36] by applying a 12-parameter affine transformation, followed by a nonlinear warping using basis functions [37]. Images were subsequently smoothed with an 8-mm isotropic Gaussian kernel and band-pass-filtered in the temporal domain.

### DTI preprocessing and analysis

DTI data were preprocessed using FSL software package (http://www.fmrib.ox.ac.uk/fsl/) [38]. Raw data were first converted to analyzable format using dcm2nii in MRIcron software (http://www.mccauslandcenter.sc.edu/mricro/mricron/index.html) developed by Dr. C. Rorden. The DTI data were first corrected for eddy currents and head motion, followed by removal of non-brain tissues. After that, a diffusion tensor model was fitted at each voxel to compute, among other measures, fractional anisotropy (FA). The FA maps created were then processed using the track-based spatial statistics routine [38] in which each individual FA map was aligned to the standard 1×1×1 mm^3^ in MNI space. These aligned FA maps were averaged to create a mean FA map, and a thinning algorithm was applied to create a mean FA skeleton that represents the centers of all fiber bundles common to all participants. After that, each participant's aligned FA map was projected onto the skeleton such that an alignment-invariant track representation of FA values was achieved for each participant.

Furthermore, to track fibers connecting different nodes within the default mode network, we used MedINRIA (http://www-sop.inria.fr/asclepios/software/MedINRIA/) software package. Tractography was done using the preprocessed data of each participant obtained from FSL. Initially, fibers were extracted as a whole over the head and then limited to specific regions. We used the following parameters to extract fibers: FA  = 200; minimum length  = 3 mm; sampling  = 1; smoothness  = 10.

### Regions of Interest (ROIs)

**Figure 1:**
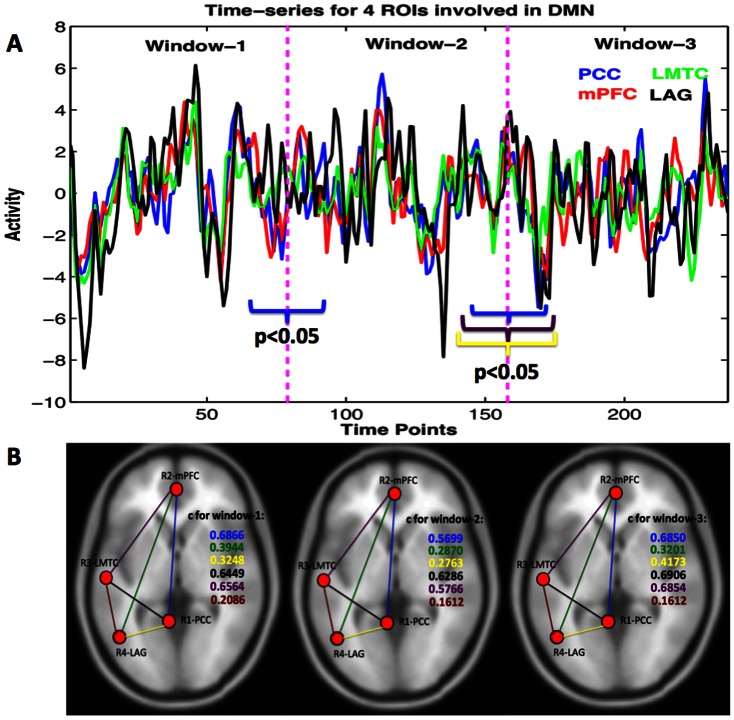
Voxel-averaged time-series for four brain regions and correlation coefficients between pairs. (A) Times-series averaged over all the trials for four nodes, and (B) positive significant correlation coefficients (c) were found to be significantly varying from one window to next.

For resting state fMRI connectivity analysis, we selected the posterior cingulate cortex (PCC) with an 8 mm radius sphere centered at (–6, –52, 40) in the MNI coordinate system as a seed region based on fMRI literature. This region is generally considered as a seed region for one of the default mode networks [23], [29], [30]. The correlated regions to the PCC were the medial prefrontal cortex (mPFC); the left middle temporal cortex (LMTC) and the left angular gyrus (LAG) (figure 1). MARSBAR software package (http://marsbar.sourceforge.net/) was used to make masks and extract BOLD time series for further time-frequency brain node and network analysis.

For DTI analysis, regions of interest (ROIs) were defined for each participant. A sphere of radius 12 mm–15 mm was drawn for each ROI with the help of the landmarks of these masks and the brain atlas.

### Time-Frequency Node and Network Activity Analysis

The node and network activities were analyzed by using the wavelet-transform based power, coherence and Granger causality techniques [25], [39]. The spectral density matrices S (t, f) were first estimated by using the direct wavelet transforms (W (t, f)) of the fMRI time series extracted from different ROIs. We used the Morlet wavelet as the mother wavelet [40]. The wavelet spectral density estimated for nodes l and m as a function of time and frequency is 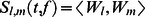, where <*> is averaging over a combination of multiple voxel time series in the nodes l and m. The diagonal (l = m) elements of the spectral density matrix (S (t, f)) represent the node activity in terms of spectral power and the network activity is derived from the off-diagonal (l≠m) elements. The coherence between node l and m is 
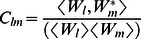
. The causal influence I(t, f) from node l to node m is computed by factorizing the spectral matrix S into minimum-phase spectral factors, deriving the transfer function H(t, f) and noise covariance matrix Σ, and using H and Σ in Granger causality formula [39]:
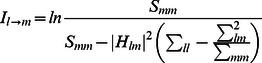
(1)


After we computed time-frequency Granger causality spectra from one node to another, we estimated the net causal flow into a node m i.e. total causality towards node m minus the total causality away from the node m, as follows: 
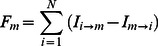
(2)where N is the total number of nodes in a network and the self-causality is assumed to be 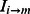 =  0 for l = m. Here, a positive F represents the net incoming information flow towards the node (sink) and a negative F refers to the net outgoing flow away from the node (source).

## Results

In this study, we computed wavelet-based time-frequency power, coherence and Granger causality spectra from fMRI BOLD time series recorded under eyes-open resting conditions, evaluated the relationships between node activity and network activity, and network activity and structural connectivity. The brain nodes included PCC, mPFC, LMTC and LAG of the default mode network.

### Dynamic nature of resting state network

**Figure 2:**
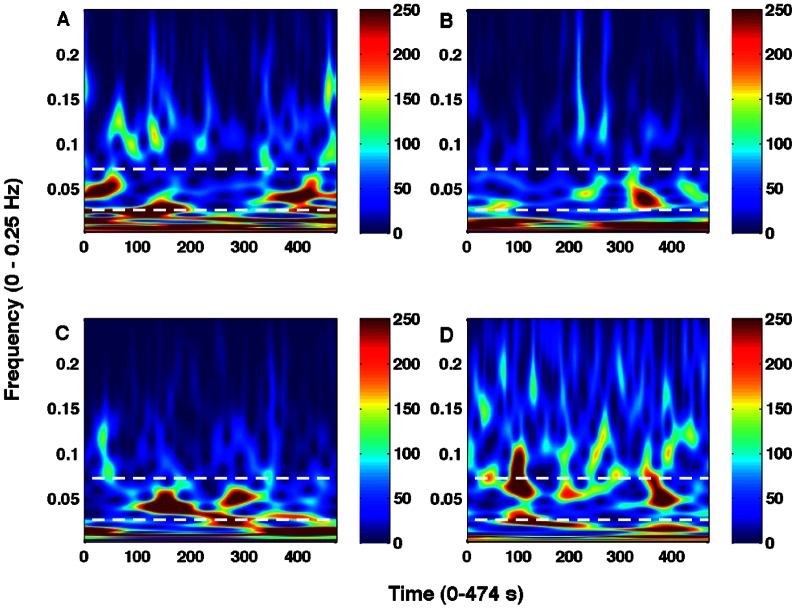
Time-frequency power spectra from a representative participant. For regions: (A) PCC, (B) mPFC, (C) LMTC, and (D) LAG, the white dotted lines marked the region for fluctuations for frequencies at slow-4.

**Figure 3:**
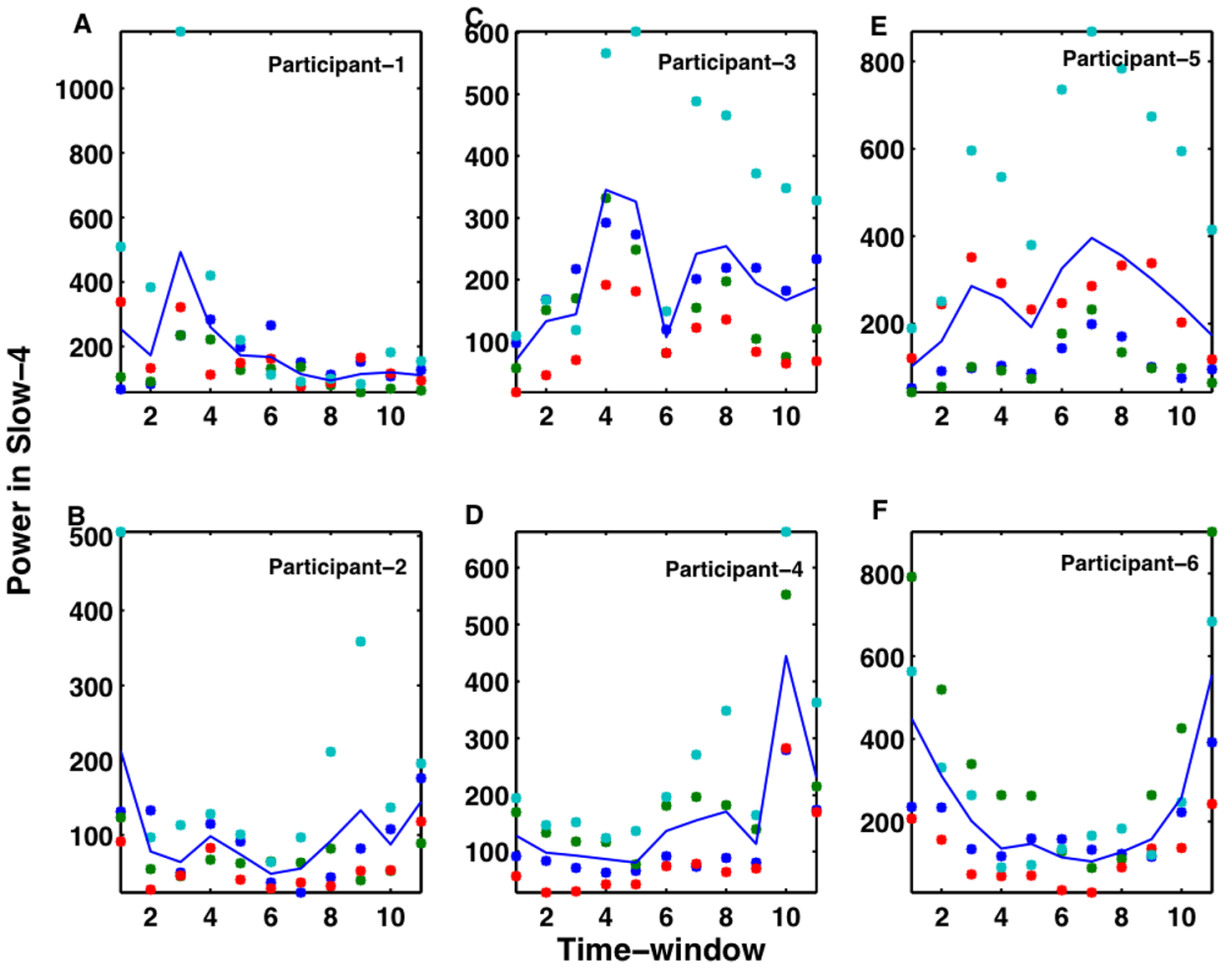
Time-varying nature of power in 6 sample participants. Slow-4 frequency band activity fluctuates significantly (A–F). Overall, there is a significant power variation across different time windows, each of size 38 sec in the slow-4 band (0.0027–0.073 Hz) for 11 out of 17 participants.

Figure 1 shows voxel-averaged time series from the four nodes and the group-level functional connectivity (correlation coefficients, c) values between pairs of these nodes over three representative time-windows. Paired t-test, underlying the hypothesis that the samples come from distributions with equal means at 5% significance level (p < 0.05), is used to determine whether there is significant difference between powers calculated over windows using a sample size of n = 79 (n = N/3 where N = 237 is the total number of scans). The functional connectivity value of one link significantly changed in going from the first window (r12 = 0.6866) to the second window (r12 = 0.5699) at p = 0.0343 (t-stat  = 2.31, sd  = 0.34). Also, several connectivity values changed in going from the second window (r23 = 0.5766, r12 = 0.5699, r14 = 0.2763) to the third window (r23 = 0.6854, r12 = 0.6850, r14 = 0.4173) at p = 0.0435 (t-stat  = –2.19, sd  = 0.34), p = 0.0340 (t-stat  = -2.31, sd  = 0.34) and p = 0.0263 (t-stat  = –2.44, sd = 0.27) respectively. Here r12, r14, r23 represent correlation coefficients from regions 1 to 2, 1 to 4 and 2 to 3 respectively and sd represents standard deviation. These results indicated that the network-level activity was dynamic. The dynamic nature of the network could be reflected in the node-level activity. To evaluate that, we computed wavelet-based time-frequency spectral map as shown in figure 2. These results of wavelet power spectra confirm that the BOLD fluctuations occur at low frequency (<0.1 Hz) oscillations and reveal further that these fluctuations might be varying in their amplitudes over time. We computed average power over different bands (slow 2 to slow 5) below 0.25 Hz for each region and each participant. We separated these frequency-averages into different time-windows; each of length 19 scans (38 seconds). One-way ANOVA within each participant was conducted to see whether the power in fixed time-windows varied significantly across time. We found that the power varied significantly in slow-4 band (0.0027–0.073 Hz) for 11 out of 17 participants with F (3, 43) ≥4.42 and p<0.009. Figure 3 shows these results from 6 representative participants, which were chosen arbitrarily out of 11 subjects whose data showed significant power variation over time. These results support that the amplitudes of resting-state node and network activity as reflected by fMRI BOLD fluctuations can change over time at frequencies below 0.1 Hz (for frequencies at slow-4).

### Node-Network Activity Relation

**Figure 4:**
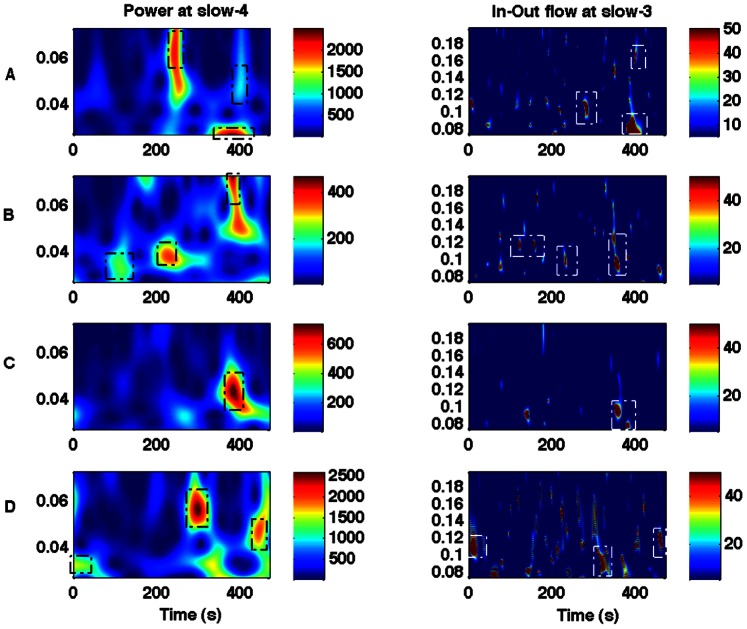
Wavelet power and in-out causal flow. Power at slow-4 frequency (left column) and in-out flow at slow-3 frequency (right column) for regions: (A) PCC, (B) mPFC, (C) LMTC, (D) LAG. Dotted boxes are used to highlight that the higher values of power and the in-out flow originate at around the same time points but at different frequencies.

**Figure 5:**
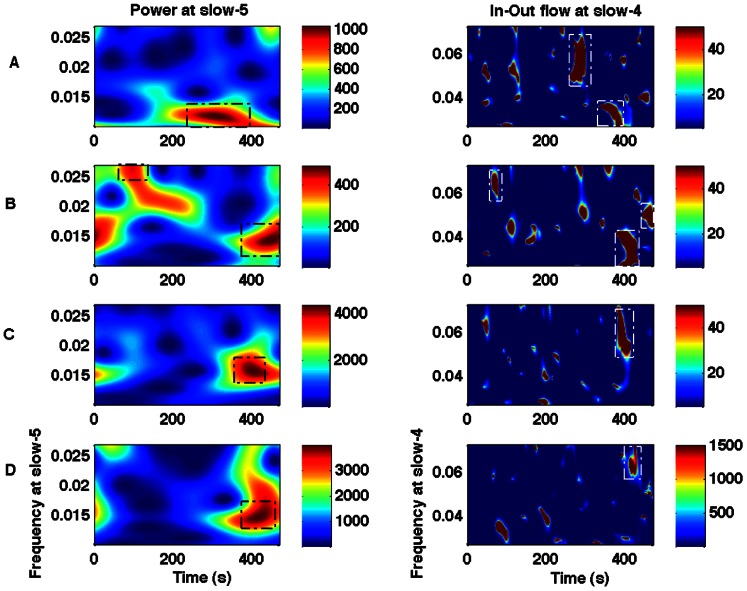
Wavelet power and in-out causal flow. Power at slow-5 frequency (left column) and in-out flow at slow-4 frequency (right column) for regions: (A) PCC, (B) mPFC, (C) LMTC, (D) LAG. Dotted boxes are used to highlight that the higher values of power and the in-out flow originate at around the same time points but at different frequencies as for the pair (slow-4 power, slow-3 net causal flow) as shown in figure 4.

We computed the wavelet power, coherence and Granger causality spectra for all four regions (PCC, mPFC, LMTC and LAG). We evaluated the relationship between the power (node activity) and the net causal flow (network activity) into each node. The net causal flow F was computed according to the definition described in Eq. (2). We found that the slow-5 node was related to the slow-4 network activity, and the slow-4 node activity was related to the slow-3 network flow. Figure 4 A–D shows, in a representative participant, the wavelet power spectra for the slow-4 frequency band and the net causal flow for the slow-3 frequency band for the nodes: PCC, mPFC, LMTC and LAG. These plots show that the peak values of power occurred around the same time where the maximum in-out flow occurred at 400 sec for PCC, 350 sec for MPFC, 362 sec for LMTC and 458 sec for LAG but at different frequencies. Similarly, figure 5A–D shows, also in a representative participant, the wavelet power spectra for the slow-5 frequency band and the net causal flow for the slow-4 frequency band for these nodes. The peak activities are marked in these plots. These plots also show that the peak values of power occurred around the same time where the maximum in-out flow occurred at 290 sec for PCC, 426 sec for MPFC, 392 sec for LMTC and 426 sec for LAG but again at different frequencies. We considered only the peak activities and computed the group level-averages for all these regions.

**Figure 6:**
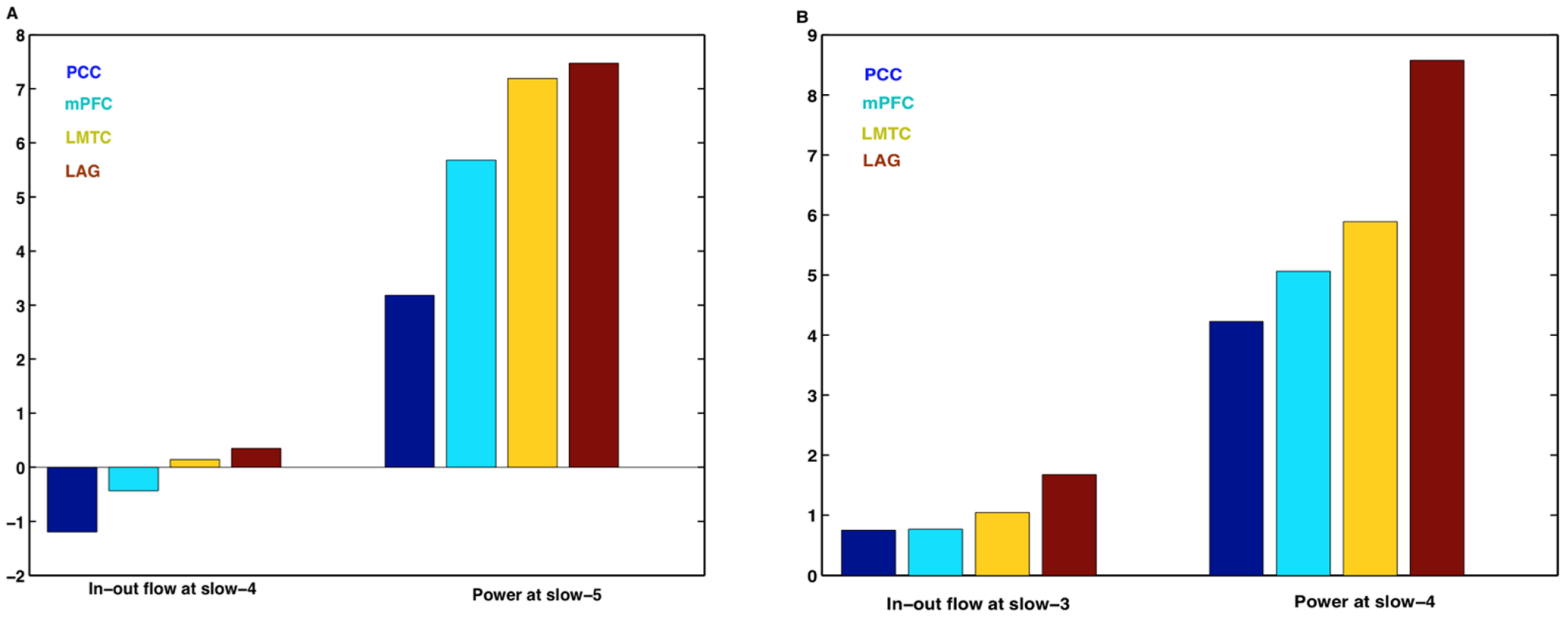
Spectral peaks of causal flow and related power at lower frequencies. (A) In-out flow at slow-4 and power at slow-5, and (B) in-out flow at slow-3 and power at slow-4. Units of power and in-out flow are arbitrary but normalized to same scale.

**Figure 7:**
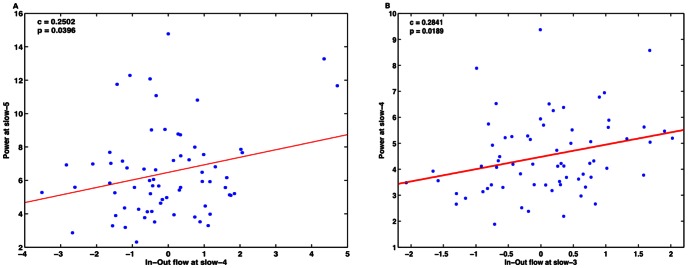
Linear relationship of the net in-out flow with power for all four nodes. (A) Slow-4 causal flow is related with the power at slow-5, (B) slow-3 causal flow is related with power at slow-4.

Figure 6A shows the slow-5 band-integrated power, averaged over participants, at each node along with the slow-4 band-integrated in-out causal flow at the corresponding node. Similarly, figure 6 B shows corresponding plots for the slow-4 and slow-3 frequency bands. A Pearson product-moment correlation coefficient is computed to assess the relationship between the peak values of in-out causal flow towards the node and the node power corresponding to same time where we have peak in-out causal flow. A significant positive linear correlation is found between the slow-4 in-out flow and the slow-5 power (c = 0.2502 at p = 0.0396, n = 68) and the slow-3 in-out flow and the slow-4 power (c = 0.2841 at p = 0.0189, n = 68) when we consider data from all four nodes in all participants (figure 7 A–B). We did not find such relations in the other combinations of in-out flow and power at same (in-out flow at slow-3, 4 and 5 frequency bands with power at slow-3, 4 and 5 frequency bands respectively) or different frequency bands (in-out flow at slow-3, 4 and 5 frequency bands with power at slow-5, 3 and 3 or 4 frequency bands respectively). Hence, we can see that the greater the net flow into a node, the higher the activity at that node at a lower frequency.

### Structure-Function Relationship

**Figure 8:**
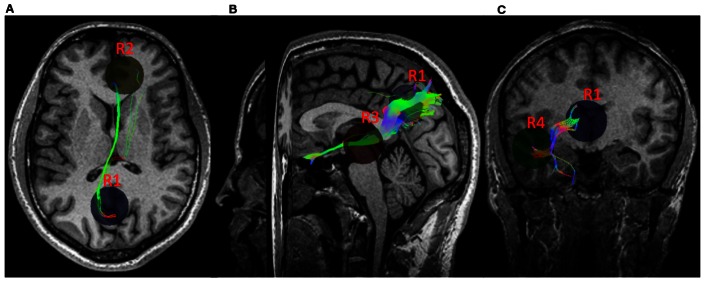
DTI fiber tracts from a representative participant. Fiber pathways between: (A) PCC (R1) and mPFC (R2), (B) PCC (R1) and LMTC (R3), and (C) PCC (R1) and LAG (R4).

**Figure 9:**
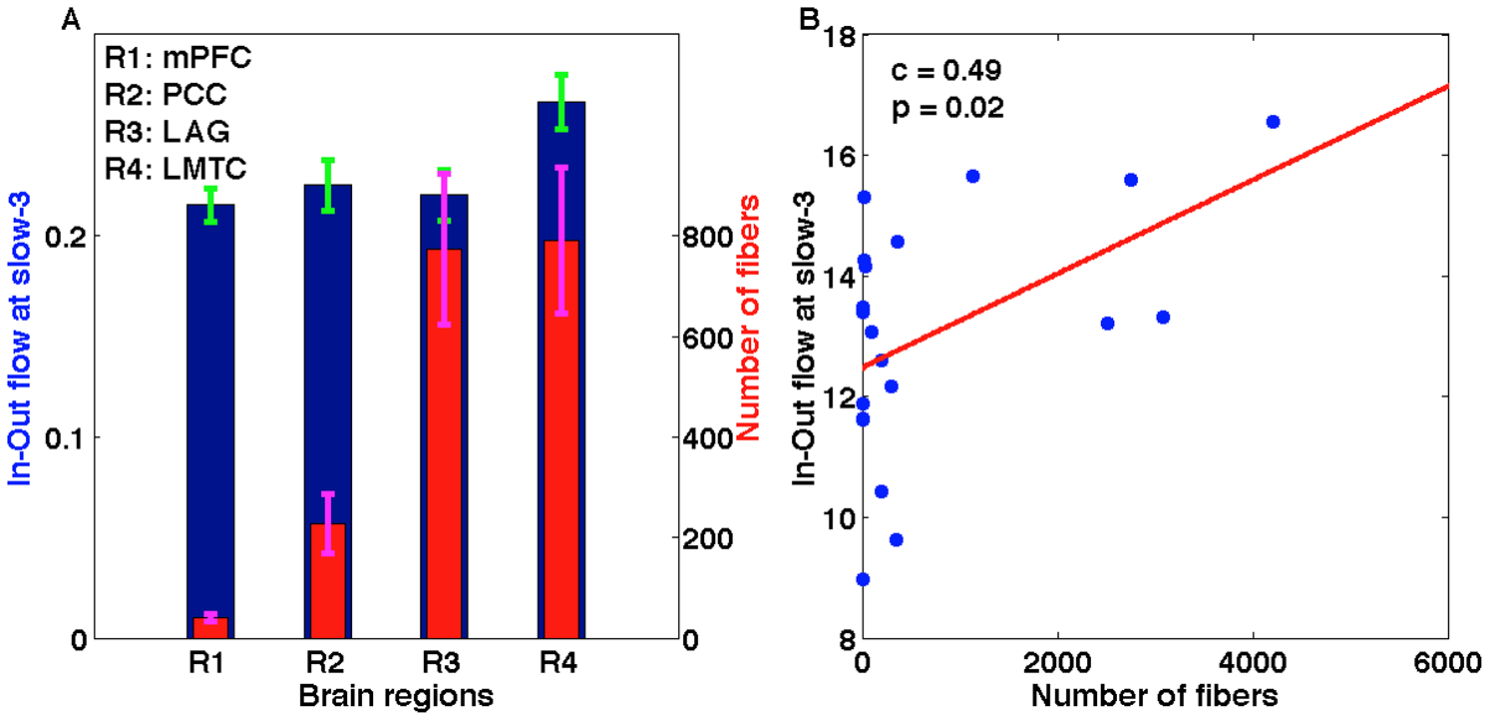
Structure-function relation. The maximum in-flow and number of fibers for all the regions have been plotted: (A) the maximum in-flow at slow-3 (blue bars) averaged over all participants for each region and corresponding number of fibers (red bars) averaged over all participants for the corresponding regions, and (B) a positive significant linear relation between the in-out flow at slow-3 and the number of fibers connecting all four regions in 6 participants who underwent both DTI and fMRI scanning.

The DTI analysis on fiber tractography using MedINRIA (http://www-sop.inria.fr/asclepios/software/MedINRIA/) showed the following structural connections to exist between network nodes: (i) R1–R2 in 25 out of 32 participants, (ii) R1–R3 in 20 out of 32, (iii) R1–R4 in 19 out of 32, (iv) R2–R3 in 18 out of 32, (v) R2–R4 in 6 out of 32, (vi) R3–R4 in 30 out of 32 participants, where R1, R2, R3, and R4 stand for the posterior cingulate cortex (PCC), medial prefrontal cortex (mPFC), left middle temporal cortex (LMTC) and left angular gyrus (LAG) respectively. We determined the number of connecting fibers to each of the four nodes from rest of the three nodes for each participant. Figure 8 shows the connecting fibers of the PCC with the other three nodes of the network. We compared the in-out flow at slow-3 frequency with the number of fibers averaged over all the participants among the regions (figure 9A). A Pearson correlation coefficient was computed to assess the relationship between the in-out causal flow towards the node and the fiber density from or to the node (figure 9 B). There was a significant correlation of c = 0.49 between the two at p = 0.02 for all the 6 common participants scanned during fMRI and DTI data collection. These results show that node activity depends on the causal flow and the causal flow in turn can show dependence on the fiber density.

## Discussion

Here in this study, we found that the low-frequency BOLD fluctuations and brain network activity during resting conditions can vary over time, but in specific lower frequency bands (slow-4, and slow-5). The lower-frequency (slow-4 and slow-5) activities in the default-mode nodes are correlated with the higher frequency (slow-3 and slow-4) network activities. The net network activity flow in slow-3 frequency band into a node is correlated with the number of DTI fiber tracts leading to the node. Our findings are confirmation and extension of the findings from three previous studies. Zuo and colleagues [6] had found that fMRI BOLD fluctuations can be reliably broken into various low-frequency bands specific to different brain regions. Chang and Glover [23] had showed that resting-state networks can vary over time. Jiao and colleagues [24] had showed that the causal information flow can predict power in a node. The non-stationary nature of functional network was also reported recently by Liu and Duyn but the oscillatory aspects of these networks were not investigated [41].

Using the nonparametric spectral approach to wavelet power, coherence, Granger causality [25], [39] we revealed the time-varying nature of low-frequency node activity and network activity. Time-varying amplitudes of oscillations occurred below 0.1 Hz. The network-level correlation and coherence were changing over time also in the frequency bands less than 0.1 Hz. It indicated that the resting state might not be always at rest. The reasons behind this unrest could be a change in behavior (awareness or arousal levels) or due to the intrinsic nature of self-organized systems like the neuronal systems in the brain [41]. A similar observation recorded from unconscious, anesthetized macaques [42] suggests that some of this non-stationarity cannot be just unconstrained mental activity. A recent publication by Smith propose the idea that automatic processes like breathing, heart rate, mind wandering and daydreaming keep some of the brain circuits active and these activities may have been dubbed the resting state [43]. Cardiac processes also cause low frequency fluctuations which may act as sources of variance in fMRI BOLD signals at resting state [44]. Situations like ‘eyes opening’ and ‘eyes closing’ are also known to affect BOLD signal fluctuations [45]. The ongoing fluctuating metabolic processes in the brain could be reasons for time-varying behaviors of the node and network activities. However, the network activity associated with respiratory related frequency (slow-2) was found to have no relationship with the node activity in any of the frequency intervals. According to Raichle and colleagues, the activity during resting state keeps the brain in an organized fashion and spontaneous activity helps to keep the neuron connections continuous with age and learning [46]. Morcom and Fletcher propose that default mode network is more active at rest than during an explicit task and there exists a physiological baseline, which can be observed when the participants are awake, but resting with eyes closed [47]. Frank and Karlsson ’s work on memory consolidation state that the activity in human brains during resting state could reactivate the patterns that correspond to past experiences [48]. These points point us to the different possibilities of how the different neural processes could be the reasons for these time-varying fluctuations. A direct positive significant correlation between these frequency pairs of network and node activity: (slow-5 node, slow-4 network) and (slow-4 node, slow-3 network) indicates that there is a structure in BOLD network and network oscillations. The low-frequency node activity can be predicted from comparatively high-frequency network activity, which signifies the importance of minute details of frequency bands in resting-state connectivity from fMRI BOLD fluctuations. Hence for each node, we predict that the higher the incoming causal influence, the higher the power of the node receiving causal information with each of the frequency bands reflecting a distinct and independent mechanism. These results are consistent with a study by Jiao and colleagues in 2011 [24]. They suggested that the relation between incoming causal influence and causal power could be interpreted in terms of brain’s metabolic activity, as the signal communication requires higher energy coupled to the information encoded by neuronal ensembles [24], [49], [50]. Our analysis showed that frequencies not only less than 0.1 Hz but also in the range 0.1–0.198 Hz play a significant role for functional connectivity. These results are also consistent with physical principles of superposition and modulation of waves.

Although the appropriateness of Granger causality techniques to fMRI time series has been debated in a study by Smith and colleagues [51], there is plenty of evidence supporting the effectiveness of Granger causality measures to estimate effective connectivity from fMRI time series [24], [52], [53], [54], [55], [56], [57], [58], [59]. As argued in the article by Wen and colleagues, we also restate that Granger causality is based on the mathematical framework that extends from the well-accepted measure coherence. In fact, the nonparametric approach to Granger causality both in the Fourier and wavelet domains [25], [39] demonstrates that Granger causality can be derived from the spectral matrix just like coherence. However, we recognize that the relationship between the Granger causality-based observables and the underlying state variables is not straightforward as with any statistical measure derived from BOLD responses.

Finally, we used DTI analysis and confirmed the relationship between structure and function and revealed further details about the relationship. We found that the main region PCC was connected to other nodes in most of the participants. The causal flow in distinct frequency slow-3 correlated with the number of fibers leading to the nodes. The fact that we were not able to find fiber pathways in some of the participants, may be due to the limitations of current DTI techniques [60].

Although we evaluated the default-mode for the above relationship, we envision that these results can be extended to other default-modes. These findings are consistent with Greicius and colleague’s findings [7], in which a positive linear relation was found between the fiber density and functional correlation. Further, the physiological origin of time-varying nature of functional dynamics during resting conditions still remains a topic for future investigations, which may require electrophysiological and behavioral data (e.g. eye movements) to be acquired concurrently with fMRI from participants at rest, sleep and/or at reduced consciousness under anesthesia.

## Conclusions

In this study, using wavelet-based spectral techniques, we analyzed time-frequency domain power and Granger causality from resting state fMRI BOLD signals. We found that the intrinsic low-frequency fMRI BOLD fluctuations vary in their amplitudes over time. The dynamic nature of the signals is reflected in the node and network activities of the default mode brain regions. The network activity at relatively higher frequencies (slow-3 and slow-4 bands) can predict lower frequency (slow-4 and slow-5) node activities. We observed a linear relationship of the net in-out causal flow to a node with the spectral power level at that node. The net in-out casual flow from a node was also related linearly with the fiber tracts density connected to the node. These findings suggest that (i) the so-called resting state may not always be at rest and there can be moments of 'ups' and 'downs' within the resting state, (ii) the lower frequency node activity can be predicted by a higher frequency network activity during up-state, and (iii) the node and network activity depends on the number of fiber tracts leading to the node.
